# KMUP-1, a GPCR Modulator, Attenuates Triglyceride Accumulation Involved MAPKs/Akt/PPARγ and PKA/PKG/HSL Signaling in 3T3-L1 Preadipocytes

**DOI:** 10.3390/molecules23102433

**Published:** 2018-09-23

**Authors:** Chung-Pin Liu, Pei-Chun Chau, Chain-Ting Chang, Li-Mei An, Jwu-Lai Yeh, Ing-Jun Chen, Bin-Nan Wu

**Affiliations:** 1 Department of Cardiology, Yuan’s General Hospital, Kaohsiung 802, Taiwan; liuchungpin@yahoo.com.tw; 2 Department of Pharmacology, Graduate Institute of Medicine, College of Medicine, Kaohsiung Medical University, Kaohsiung 807, Taiwan; b9220@hotmail.com (P.-C.C.); a99443@hotmail.com (C.-T.C.); anlm@kmu.edu.tw (L.-M.A.); jwulai@kmu.edu.tw (J.-L.Y.); ingjun@kmu.edu.tw (I.-J.C.); 3 Department of Medical Research, Kaohsiung Medical University Hospital, Kaohsiung 807, Taiwan; 4 Department of Medical Education and Research, Pingtung Christian Hospital, Pingtung 900, Taiwan; 5 Lipid Science and Aging Research Center, Kaohsiung Medical University, Kaohsiung 807, Taiwan

**Keywords:** protein kinases, MAPK, phosphorylated HSL, PPARγ, adipogensis, lipolysis

## Abstract

Xanthine-based KMUP-1 was shown to inhibit phosphodiesterases (PDEs) and modulate G-protein coupled receptors (GPCRs) to lower hyperlipidemia and body weight. This study further investigated whether KMUP-1 affects adipogenesis and lipolysis in 3T3-L1 preadipocytes. KMUP-1 (1–40 µM) concentration-dependently attenuated Oil Red O (ORO) staining and decreased triglyceride (TG) accumulation, indicating adipogenesis inhibition in 3T3-L1 cells. In contrast, the β-agonist ractopamine increased ORO staining and TG accumulation and adipogenesis. KMUP-1 (1–40 µM) also reduced MAPKs/Akt/PPARγ expression, PPARγ1/PPARγ2 mRNA, and p-ERK immunoreactivity at the adipogenesis stage, but enhanced hormone sensitive lipase (HSL) immunoreactivity at the lipolysis stage. Addition of protein kinase A (PKA) or protein kinase G (PKG) antagonist (KT5720 or KT5728) to adipocytes did not affect HSL immunoreactivity. However, KMUP-1 did increase HSL immunoreactivity and the effect was reduced by PKA or PKG antagonist. Simvastatin, theophylline, caffeine, and sildenafil, like KMUP-1, also enhanced HSL immunoreactivity. Phosphorylated HSL (p-HSL) was enhanced by KMUP-1, indicating increased lipolysis in mature 3T3-L1 adipocytes. Decreases of MAPKs/Akt/PPARγ during adipogenesis contributed to inhibition of adipocyte differentiation, and increases of PKA/PKG at lipolysis contributed to HSL activation and TG hydrolysis. Taken together, the data suggest that KMUP-1 can inhibit hyperadiposity in 3T3-L1 adipocytes.

## 1. Introduction

The lipids-related properties of the xanthine derivative KMUP-1 have been demonstrated in vivo in animal models. KMUP-1 improved hepatic lipid metabolism and steatohepatitis in high-fat diet (HFD) mice [1,2]. Whether KMUP-1 affects lipid metabolism in 3T3-L1 preadipocytes has not yet been explored. This study examined whether KMUP-1 inhibits adipogenesis and promotes lipolysis via protein kinase A (PKA) and protein kinase G (PKG) in 3T3-L1 cells. Functional studies and radioligand binding assays of G-protein couple receptors (GPCRs) in CHO-K1 cells revealed that KMUP-1 (7-[2-[4-(2-chlorophenyl)piperazinyl]ethyl]-1,3-dimethylxanthine) inhibited phosphodiesterases (PDEs)-dependent cAMP/cGMP degradation and attenuated β-adrenoceptor agonist-induced cardiac hypertrophy in cardiovascular systems [3,4].

3T3-L1 cells possess a fibroblast-like morphology and can differentiate into an adipocyte-like phenotype under proper situations [5]. 3T3-L1 preadipocytes are sensitive to lipogenic and lipolytic hormones and drugs, including insulin and β-adrenoceptor agonists [6]. The development of mature adipocytes from precursor fat cells designated as preadipocytes [7], includes proliferation of preadipocytes, fat cell differentiation (adipogenesis) lipolytic activity, and apoptosis of preadipocytes or mature adipocytes [7]. In this study, we explored KMUP-1 modulation of adipogenesis and lipolysis in the life-cycle of 3T3-L1 preadipocytes in hyperadiposity.

Inhibition of adipogenesis and lipolysis interferes with adipocyte maturation. Adipogenesis is controlled by activation of protein kinases, such as PKA/PKG and mitogen-activated protein kinases (MAPKs; ERK/p38/JNK) in adipocytes [6,7,8]. Lipolysis occurs in all tissues and cell types, is most abundant in white and brown adipose tissues and is the biochemical pathway responsible for the catabolism of triglyceride (TG) [9] stored in cellular lipid droplets. The hydrolysis of TG generates non-esterified fatty acids, which are used as energy substrates [7,8]. Additionally, enhanced PKA phosphorylates perilipin on the droplets, resulting in lipolysis by HSL/p-HSL [10].

Phosphodiesterase type 5 (PDE5) inhibitors affect lipid metabolism by increasing cGMP-dependent PKG. Sildenafil, a specific PDE5 inhibitor, promotes adipogenesis accompanied by increasing glucose uptake through a PKG pathway in 3T3-L1 cells [11]. Chronic sildenafil treatment improves energy balance and enhances in vivo insulin action in an HFD mouse model [12]. Notably, the unique PDE inhibitor KMUP-1, which activates PKA and PKG, partly shares a similar mechanism of action in 3T3-L1 preadipocytes. Additionally, PPARγ, a regulator of lipid metabolism, is expressed in 3T3-L1 preadipocytes [9,13]. Inhibition of PDE5 and PDE3B induce adipocyte lipolysis by cGMP/PKG and cAMP/PKA elevation [14,15]. HSL regulates the hydrolysis of acylglycerol and cholesteryl ester in various cells and organs, including adipose tissues. HSL is phosphorylated by PKA and PKG to increase the lipolysis of adipocytes [16].

GPCRs-mediated kinase pathways, including MAPK/ERK and MEK1 via allosteric receptor binding, have become increasingly important for the identification of GPCR agonists or antagonists targeting adipogenesis and/or lipolysis [17,18]. Xanthine-based caffeine has also been proposed to have several beneficial effects on obesity, especially on energy expenditure and thermogenesis in brown adipose tissues (BATs) through uncoupling protein 1 (UCP-1) and adrenergic activation [19], characterized by activation of hormone sensitive lipase (HSL) at adipocytes [20,21]. In this regard, the effects of KMUP-1 remain undetermined in 3T3-L1 preadipocytes. Thus, we determined the effects of the GPCR modulator KMUP-1 on adipogenesis and lipolysis proteins, with immunofluorescent staining of 3T3-L1 cells to reveal possible anti-obesity mechanisms.

## 2. Results

### 2.1. KMUP-1 and Ractopamine Affected the Adipocyte Development

**Figure 1:**
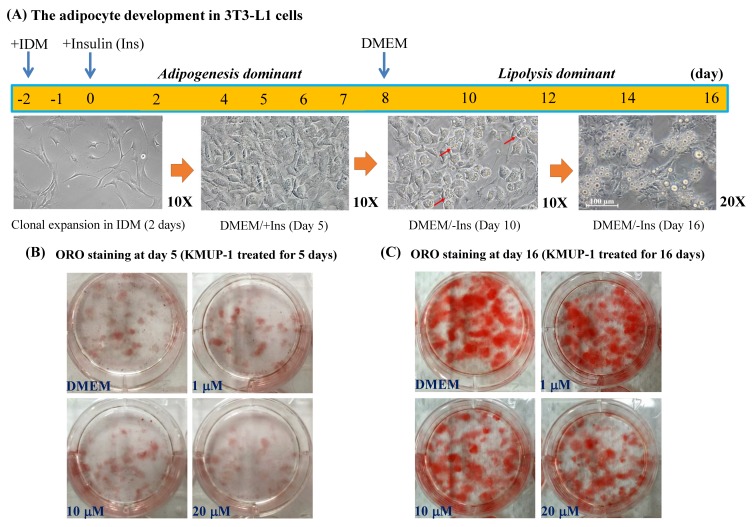
Demonstration of the adipocyte development and ORO staining of lipid accumulation in 3T3-L1 cells. (**A**) Fibroblast cells achieved mitotic clonal expansion with IDM for 2 days. 3T3-L1 preadipocytes cultured in DMEM medium were differentiated in the presence (Day 0 to Day 7) and absence (Day 8 to Day 16) of insulin. (**B**) ORO staining at Day 5. KMUP-1 (1, 10, and 20 µM) reduced oil-droplet accumulation (red color), implying adipogenesis involved. (**C**) ORO staining at Day 16. KMUP-1 decreased lipid accumulation (red color), implying lipolysis involved. Red arrow indicates the oil droplet in adipocytes.

As shown in Figure 1A, 3T3-L1 preadipocytes were cultured in DMEM + IDM medium for two days to induce differentiation and development of adipocytes from fibroblast cells. At Day 5, in the presence of insulin (Ins) (DMEM + Ins), the amount of ORO staining was decreased by KMUP-1 (10, 20 µM), indicating the decrease of adipogenesis or lipid accumulation in adipocytes in comparison with control (DMEM) (Figure 1B). 3T3-L1 preadipocytes do not have lipolytic activity until they are differentiated into mature adipocytes [22]. At Day 16, after removal of insulin for eight days, the oil droplets were obvious in mature 3T3-L1 adipocytes, so the period from Day 8 to Day 16 is considered as the stage of lipolysis. The quantity of ORO staining was concentration-dependently decreased by KMUP-1 (1–20 µM), indicating stimulation of lipolysis and/or reduction of lipid accumulation in adipocytes (Figure 1C).

### 2.2. ORO Staining, TG Content, and Cell Viability

**Figure 2:**
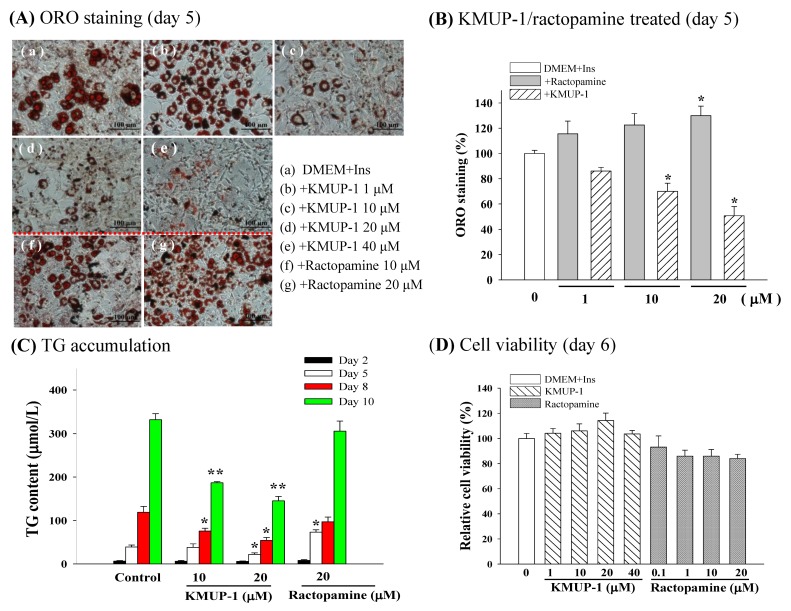
Effects of KMUP-1 (1–40 µM) and ractopamine (10, 20 µM) on ORO staining in 3T3-L1 cells, TG accumulation, cell viability at Day 5 after differentiation. (**A**) The concentration-dependent effect of KMUP-1 (b–e) and ractopamine (f,g) on ORO staining in DMEM + insulin (Ins) culture medium. (**B**) Data from ORO staining were analyzed at Day 5 after differentiation. (**C**) KMUP-1 (10, 20 µM) and ractopamine (20 µM) reduced and increased TG accumulation, respectively. (**D**) Cell viability was not affected by KMUP-1 (1–40 µM) or ractopamine (0.1–20 µM). * *p* < 0.05, ** *p* < 0.01 indicates significant (n = 8).

At Day 5, the control group (DMEM + Ins) significantly increased the lipid accumulation in differentiated adipocytes (Figure 2A, a). Treatment for 5 days of KMUP-1 (1–40 µM) concentration-dependently attenuated the amount of ORO staining, indicating the inhibition of adipogenesis (Figure 2A, b–e). In contrast, the β-adrenergic agonist ractopamine increased the ORO staining, indicating the stimulation of adipogenesis (Figure 2A, f,g). Obtained results analysis in Figure 2B, KMUP-1 (10, 20 µM) and ractopamine (20 µM) significantly reduced and increased the ORO staining, respectively. As shown in Figure 2C, 10 and 20 µM KMUP-1 time-dependently decreased the accumulation of TG levels in adipocytes at Day 8–10 and Day 5–10, respectively, in comparison with control. However, ractopamine (20 µM) increased the accumulation of TG levels in adipocytes at Day 5. Meanwhile, both KMUP-1 (1–40 µM) and ractopamine (0.1–20 µM) appeared not to affect the viability of 3T3-L1 cells using the MTT test (Figure 2D).

### 2.3. KMUP-1 Affected MAPKs/p-Akt/PPARγ Expression

**Figure 3:**
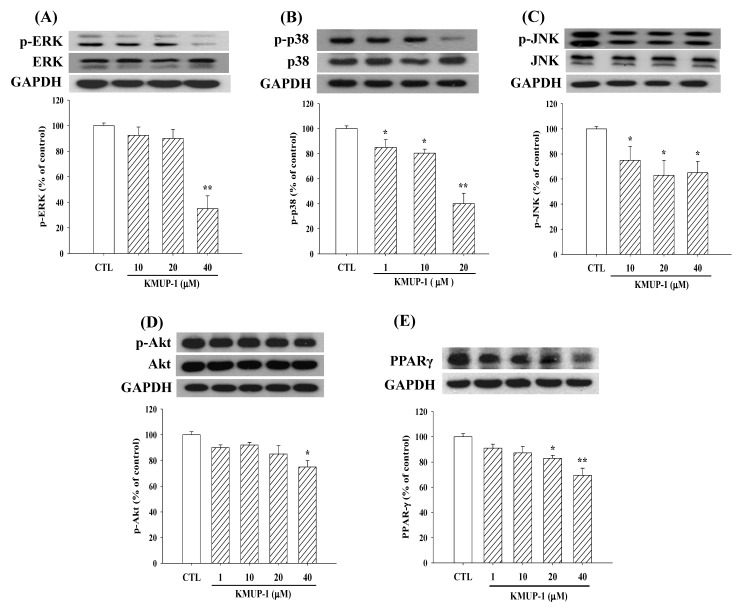
Effects of KMUP-1 (1–40 µM) on p-ERK/p-p38/p-JNK/p-Akt/PPARγ expression in 3T3-L1 cells at Day 5 after differentiation. Protein expression was assessed as described in detail in the Materials and Methods section. Data are means ± S.E., n = 8 in each group. * *p* < 0.05, ** *p* < 0.01 versus control (CTL) group. CTL: DMEM + insulin.

We observed some adipogenesis-related proteins in the differentiation process at Day 5. KMUP-1 (1–40 µM) inhibited p-ERK/p-p38/p-JNK, p-Akt, and PPARγ protein expression in 3T3-L1 cells (Figure 3). Notably, KMUP-1 seemed more sensitive to phosphorylated p38 expression in the MAPK proteins with supplemented insulin in the culture medium.

### 2.4. KMUP-1 Influenced PPARγ1/PPARγ2 mRNA and p-ERK Immunoreactivity

**Figure 4:**
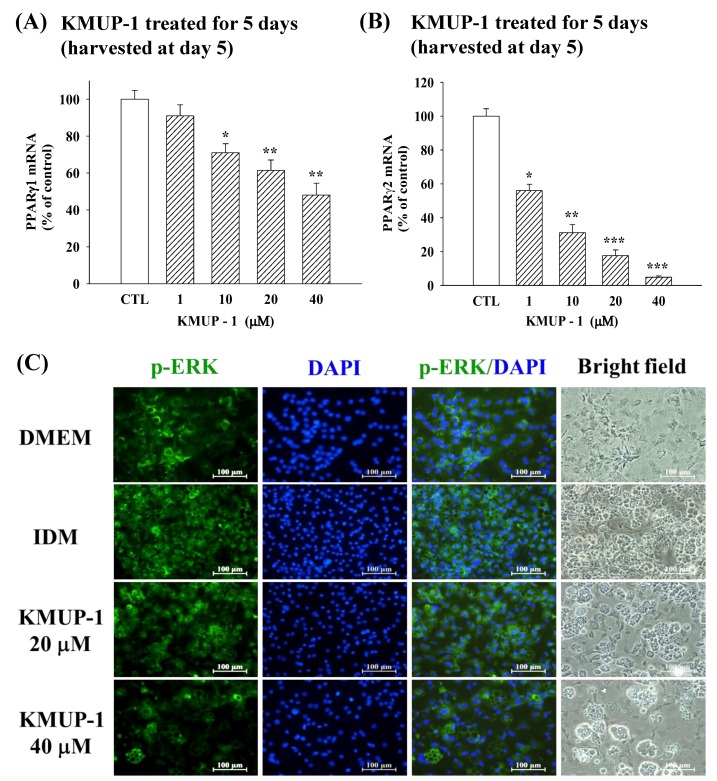
Effects of KMUP-1 on PPARγ1/PPARγ2 mRNA and p-ERK immunofluorescence in 3T3-L1 cells at Day 5. The mRNA of PPARγ1 (**A**) or PPARγ2 (**B**) was assayed by qPCR as described in detail in the Materials and Methods section. Data are means ± S.E. of three independent experiments and expressed as relative value to control. * *p* < 0.05, ** *p* < 0.01 versus control (CTL) group (n = 8). CTL: DMEM + insulin. (**C**) DMEM, IDM, and KMUP-1 were used to estimate p-ERK immunoreactivity. DAPI was used for staining nucleus in blue. Scale bar: 100 µm.

In 3T3-L1 cells after 2 days’ differentiation, KMUP-1 (1–40 µM) was added to adipocytes for 5 days and the expression of PPARγ1 or PPARγ2 mRNA was concentration-dependently decreased (Figure 4A,B). It appears that KMUP-1 was more sensitive to PPARγ2 mRNA than PPARγ1 in the adipogenesis stage. The immunofluorescence of phosphorylated ERK was measured using confocal microscopy. KMUP-1 (20, 40 µM) attenuated the p-ERK immunoreactivity at Day 5, in comparison with IDM culture medium (Figure 4C). This suggested that KMUP-1 attenuated adipogenesis in 3T3-L1 cells.

### 2.5. KMUP-1 Influenced HSL/p-HSL Immunoreactivity

**Figure 5:**
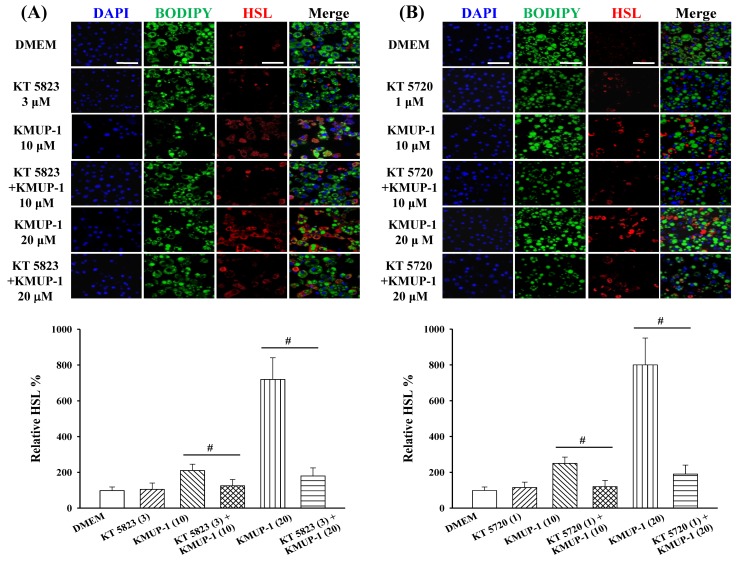
Immunofluorescence of HSL in 3T3-L-1 cells in the presence and absence of PKG and PKA antagonists (KT5823 and KT5720) at Day 8 and treatment with KMUP-1 for 2 days. (**A**) Cells were incubated with DMEM, KT 5823 (3 µM), KMUP-1 (10 and 20 µM), and KT 5823 + KMUP-1 to estimate HSL immunoreactivity. The bar chart indicates the percentage changes of relative HSL. (**B**) Cells were incubated with DMEM, KT 5720 (1 µM), KMUP-1 (10 and 20 µM), and KT 5720 + KMUP-1 to estimate HSL immunoreactivity. The bar chart indicates the percentage changes of relative HSL. Data are means ± S.E. of three independent experiments and expressed as relative value to DMEM. ^#^ *p* < 0.05 versus KT 5823 + KMUP-1 or KT 5720 + KMUP-1 group. DAPI was used for staining nucleus in blue. Scale bar: 60 µm.

To prevent possible interference from KMUP-1 in lipolysis, the mature of 3T3-L1 adipocytes were cultured in DMEM only from Day 8 to Day 16. At Day 8 and treatment with KMUP-1 (10–20 µM, 2 days) concentration-dependently facilitated the translocation of HSL from the cytosol to membrane. Notably, KMUP-1-stimulated HSL immunoreactivities were significantly inhibited by PKG and PKA inhibitors (KT 5823 and KT 5720), suggesting that KMUP-1’s effect can be attributed to PKG and PKA activation. By contrast, neither KT 5823 nor KT 5720 alone influenced the basal HSL immunoreactivity (Figure 5A,B).

**Figure 6:**
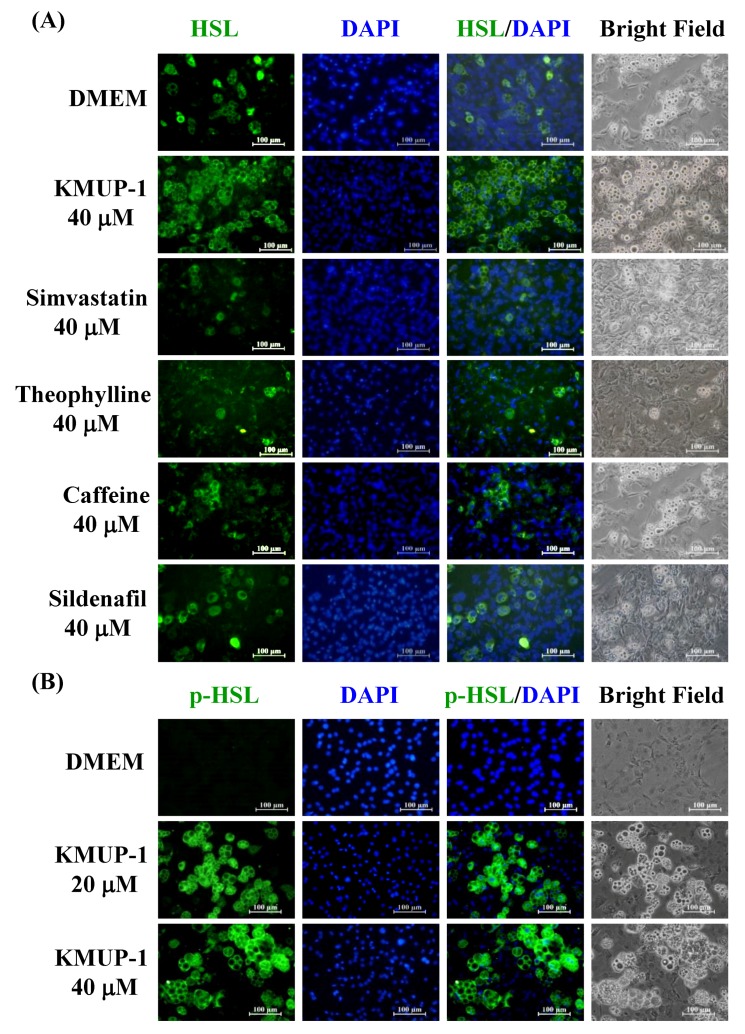
Immunofluorescence of HSL or p-HSL in 3T3-L1 adipocytes incubated with KMUP-1, simvastatin, theophylline, caffeine, or sildenafil. (**A**) DMEM and KMUP-1/simvastatin/theophylline/caffeine/sildenafil at 40 µM were used to observe HSL immunoreactivity. (**B**) KMUP-1 (20 and 40 µM) also affected p-HSL immunoreactivity. DAPI was used for staining nucleus in blue. Scale bar: 100 µm.

At Day 16, KMUP-1 (40 µM) and simvastatin/theophylline/caffeine/sildenafil (40 µM) increased the HSL immunoreactivity of 3T3-L1 adipocytes. KMUP-1 appeared to have the strongest HSL immunofluorescence compared to the other four agents, suggesting that KMUP-1 had the most potent effect on lipolysis (Figure 6A). KMUP-1 (40 µM) also increased the immunoreactivity of phosphorylated HSL (p-HSL), indicating the activation of HSL by KMUP-1 (Figure 6B).

### 2.6. Expression of PKA/PKG and p-HSL

**Figure 7:**
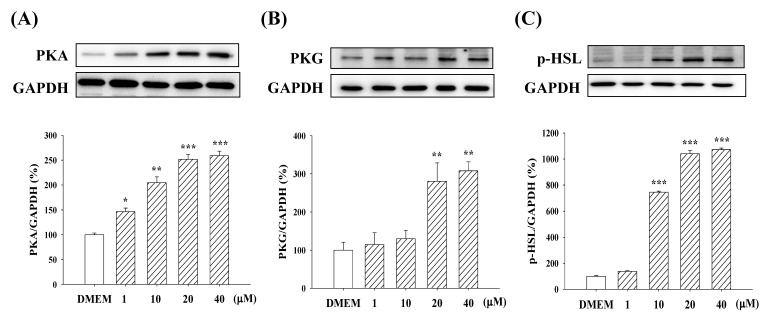
Expression of PKA, PKG, and p-HSL in 3T3-L1 adipocytes at Day 16 and treatment with KMUP-1 (1–40 µM). The protein expression was assessed as described in the Materials and Methods section. Data are means ± S.E. of three independent experiments and expressed as relative value to DMEM. * *p* < 0.05, ** *p* < 0.01, *** *p* < 0.001 versus DMEM group (n = 8).

At Day 16, KMUP-1 (1, 10, 20, and 40 µM) concentration-dependently increased the expression of PKA and PKG in 3T3-L1 adipocytes (Figure 7A,B). KMUP-1 was more sensitive to PKA protein than to PKG. Additionally, KMUP-1 also increased the expression of p-HSL, a key enzyme regulating the lipolysis process (Figure 7C).

## 3. Discussion

This study explored for the first time whether KMUP-1 modulates adipogenesis and lipolysis in 3T3-L1 adipocytes. KMUP-1 was found to reduce ORO staining and decrease TG accumulation in 3T3-L1 adipocytes. At the adipogenesis dominant stage, KMUP-1 inhibited MAPKs and p-Akt/PPARγ proteins, suggesting the inhibition of adipocyte proliferation and differentiation. KMUP-1 also decreased PPARγ1 and PPARγ2 mRNA and was more sensitive to PPARγ2. At the lipolysis dominant stage, KMUP-1 enhanced HSL/p-HSL immunoreactivity, suggesting the stimulation of adipocyte lipolysis. This lipolytic effect was reduced by PKA and PKG antagonists. We thus provide evidence that KMUP-1 may inhibit adipogenesis through MAPKs/Akt/PPARγ signaling and stimulate lipolysis through PKA/PKG-dependent HSL phosphorylation.

Inhibition of adipogenesis and TG accumulation in 3T3-L1 adipocytes can be beneficial for the control of obesity. It is widely accepted that insulin-induced adipocyte differentiation, adipogenesis, and TG accumulation involves the expression of MAPKs (ERK/p38/JNK) and Akt/PPARγ in 3T3-L1 adipocytes. ERK activity is necessary for the expression of the key adipogenic regulators C/EBPα, β, and δ, and PPARγ. ERK phosphorylation of C/EBP β activates its transcriptional activity in 3T3-L1 cells [23]. Engelman et al. published the first report to describe the positive role of p38 in adipogenesis and its relation to C/EBP β phosphorylation [24]. JNK1 and the scaffold protein JNK interacting protein 1 (JIP1) are involved in the development of obesity [25]. However, one review article suggested that the blockade of p38 inhibits adipocyte differentiation, but not the ERK or JNK blockade [14]. The role of MAPKs in adipocyte differentiation and obesity still needs further investigation. Akt mediates the signaling pathway of insulin or insulin-like growth factor 1 (IGF-1) in adipogenesis [26]. Akt was also proved to mediate the antilipolytic action of insulin through phosphorylation of PDE3B, resulting in increased hydrolysis of cAMP [27]. PPARγ is a regulator of adipogenesis, expressed in adipocyte differentiation, fat storage, and inflammation [28,29]. PPARγ has two isoforms, PPARγ1 and PPARγ2. PPARγ1 is expressed in adipose and many other tissues, but PPARγ2 is restricted to adipose tissue [30]. In this study, we also found that KMUP-1 was more sensitive to PPARγ2 mRNA in adipogenesis. At the adipogenesis stage of 3T3-L1 cells, KMUP-1 attenuated the MAPKs and Akt/PPARγ signaling pathways, suggesting it has diverse activities that prevent insulin and agonist-induced adipogenesis. In contrast, the β-agonist ractopamine increased adipogenesis. Ractopamine (1, 10, and 20 µM) dose-dependently increased ORO staining and TG accumulation in the adipogenesis of 3T3-L1 cells, but we did not further measure its lipolytic activity in this study. Ractopamine has been described to produce more lean meat and less fat in animals via β-receptor agonist activity [31].

Modulation of lipolysis is an essential function in adipocyte metabolism, and altered lipolysis may contribute to fat deposition and/or obesity. It is generally agreed that both cAMP and cGMP are involved in adipose differentiation and lipolysis in adipocytes [14]. A previous study also confirmed that lipolytic mechanisms involve PKA and PKG dependent pathways, associated with subsequent fatty acid release via fatty acid-binding protein 4 (FABP4) and glycerol release via Aquaporin-7 [32]. Since adipocytes express functional PDE3B and PDE5, fat metabolism could be modulated by PDE inhibitors. Indeed, the inhibition of adipocyte PDE3B and PDE5, respectively, enhanced the intracellular cAMP/PKA and cGMP/PKG levels, and can activate HSL to stimulate lipolysis [1,2,14]. Our HSL immunoreactivity data suggest that the lipolytic activity of theophylline, caffeine, or sildenafil is less than that of KMUP-1. For lipolysis, both theophylline and caffeine mainly increased cAMP-dependent PKA, and the PDE5 inhibitor sildenafil largely increased cGMP-dependent PKG. However, KMUP-1 combined PDE inhibition and PKA/PKG activation [1,2], so it is not surprising that KMUP-1 had better lipolytic effects in adipocytes.

HSL and adipose triglyceride lipase (ATGL) are the major enzymes in adipose tissue contributing to the catabolism of TG. Phosphorylation of HSL acts together with ATGL to accelerate the lipolytic process [33]. Lipolytic hormones such as catecholamine and ACTH stimulate cAMP-dependent PKA, which phosphorylates HSL and perilipin in adipocytes. Upon lipolytic stimulation, HSL translocated from cytosol to fat droplets; conversely, perilipin moved from fat droplets to cytosol [33,34]. Likewise, increases of intracellular cGMP activate PKG, which in turn phosphorylates HSL in a similar manner. Once activated, HSL hydrolyzes the TGs in non-esterified fatty acid and glycerol [1,2,35]. Taken together, KMUP-1 enhances PKA/PKG mediated p-HSL activation, suggesting that it has potent lipolytic action in adipocytes.

**Figure 8:**
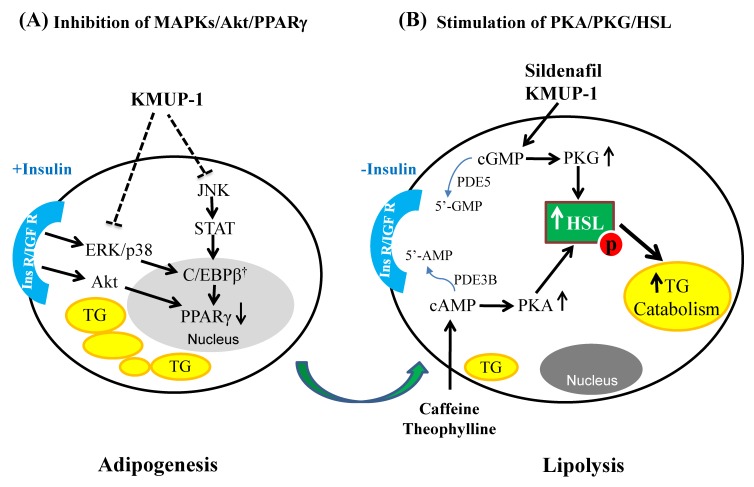
Proposed mechanism of action of the GPCR modulator KMUP-1 on the adipocyte development in 3T3-L1 cells. KMUP-1 reduces adipogenesis by inhibiting insulin-mediated MAPKs and the p-Akt/PPARγ signaling pathway and induces lipolysis by activating PKA/PKG mediated p-HSL activation, which stimulates the catabolism of TGs. ^†^ the adipogenesis gene is not measured.

In conclusion, the GPCR modulator KMUP-1 prevents lipid accumulation in 3T3-L1 adipocytes via PDE inhibition, inhibits adipogenesis via MAPKs/Akt/PPARγ signaling, and enhances lipolysis via PKA/PKG/HSL signaling pathways to reduce adiposity-based chronic disease (Figure 8). Since the relevant enzyme of ATGL and MGL had not yet been determined in the lipolysis experiments, so we acknowledge that there remain some limitations in this study. Lastly, this in vitro investigation of inhibiting adipogenesis and promoting lipolysis in 3T3-L1 adipocytes suggests that KMUP-1 could be developed as a potential pharmacotherapeutic agent for overweight or obese individuals.

## 4. Materials and Methods

### 4.1. Cell Culture

Murine 3T3-L1 preadipocytes were purchased from the American Type Culture Collection (ATCC, Manassas, VA, USA). Cells were cultured in 10% DMEM (Dulbecco’s Modified Eagle’s Medium, Invitrogen-Gibco, Carlsbad, CA, USA), 10% DMEM + insulin (10 µg/mL) or DMEM + IDM (10 µg/mL insulin, 1 µM dexamethasone, 0.5 mM 3-isobutyl-1-methylxanthine (IBMX); Sigma-Aldrich, St Louis, MO, USA), supplemented with 25 mM glucose, 10% FBS, 100 U/mL penicillin, and 100 μg/mL streptomycin. Cells were grown in a humidified atmosphere containing 95% air + 5% CO_2_ at 37 °C. After reaching confluence, cells were differentiated by incubation for 2 days in 10% DMEM + IDM, followed by 6 days in 10% DMEM + 10 µg/mL insulin, and an additional 8 days in 10% DMEM only. In general, 90% of the 3T3-L1 preadipocytes can be differentiated into mature adipocytes. To examine the role of HSL on oil droplets of adipocytes, IDM and insulin were removed from the culture medium.

### 4.2. Oil Red O Staining, TG Content and Cell Viability

At Day 5 and Day 16 of the experimental protocol, 3T3-L1 cells were washed three times with PBS and then fixed with 4% paraformaldehyde for 2 min. Oil Red O [10] solution (0.5% in isopropanol) was diluted with distilled water (3:2) filtered through a 0.45 m filter and incubated with the cells for 1 h at room temperature. Cells were washed with distilled water and the stained fat droplets in the cells were visualized by light microscopy and photographed. The percentage of differentiated cells was determined by counting cells based on ORO staining in the oil droplets. The ORO staining was analyzed by a spectrophotometer at 492 nm.

For measurement of TG content, cells were incubated with test sample for 72 h, collected, and lysed in lysis buffer (1% Triton X-100 in PBS) for 30 min, and TG content was then determined using a commercial assay kit (Zenbio, Inc., Research Triangle Park, NC, USA). The TG assay kit comprises Reagent A, Reagent B, glycerol standard (10 mM), and diluent. Readings occurred at 540 nm using a microtiter plate reader, and absorbance values were recorded as glycerol readings. The increase in absorbance at 540 nm is directly proportional to glycerol (and TG) concentration of the sample, so TG levels were calculated from the standard curve.

MTT assay protocol utilizes a commercial kit (Abcam, London, UK) to validate cell viability. MTT (0.5 mg/mL) is taken up by viable 3T3-L1 cells after 4 h incubation. The culture medium was then removed, and cells were dissolved in isopropanol and shaken for 10 min. The amount of MTT formazan (blue color) was quantified using a plate reader at 540 and 630 nm. The cell viability was calculated as follows: viability (%) = (*OD*_540, sample_ − *OD*_630, blank_)/(*OD*_540, control_ − *OD*_630, blank_) × 100.

### 4.3. Western Blotting Analysis

After 5 days of culture, 3T3-L1 cells were placed in an extraction buffer (Tris 10 mM, pH 7.0, NaCl 140 mM, PMSF 2 mM, DTT 5 mM, NP-40 0.5%, pepstatin A 0.05 mM, and leupeptin 0.2 mM) for protein extraction, and centrifuged at 12,500 *g* for 30 min. To measure protein expression levels, the total cell proteins were extracted after incubation with KMUP-1 (1, 10, 20, or 40 µM) and then Western blotting analyses were performed as described previously [1,2]. Briefly, the protein extract was boiled to a ratio of 4:1 with sample buffer (Tris 100 mM, pH 6.8, glycerol 20%, SDS 4%, and bromophenol blue 0.2%). Electrophoresis was performed using 10% SDS-polyacrylamide gel (1 h, 100 V, 40 mA, 20 µg protein). Separated proteins were transferred to polyvinylidene fluoride (PVDF) membranes treated with 5% fat-free milk powder to block nonspecific IgGs (90 min, 100 V) and incubated for 1 h with specific protein antibody. The blot was then incubated with anti-mouse or anti–goat IgG linked to alkaline phosphatase (1:1000) for 1 h. Immunoreactive bands were visualized using horseradish peroxidase–conjugated secondary antibodies with subsequent enhanced chemiluminescence (ECL) detection (GE Healthcare Bio-Sciences Corp., Piscataway, NJ, USA). Mouse or rabbit primary antibodies of p-ERK, p-p38, p-JNK, p-Akt, p-HSL (1:1000 dilution; Cell Signaling, Boston, MA, USA), PPARγ (1:1000 dilution; Santa Cruz Biotechnology, Santa Cruz, CA, USA), PKA, PKG (1:1000 dilution; Abcam, London, UK), and internal control glyceraldehyde 3-phosphate dehydrogenase (GAPDH) (1:2000 dilution; Millipore, Temecula, CA, USA) were used in Western blot analyses.

### 4.4. Real-Time Quantitative Polymerase Chain Reaction (qPCR)

mRNAs of PPARγ1 and PPARγ2 were evaluated by the qPCR method in incubation with KMUP-1 (1, 10, 20, and 40 µM) over different time periods. Total RNA was extracted from 3T3-L1 cells using RNeasy (Qiagen, Hilden, Germany). The quality and quantity of the RNA were determined by measuring absorbance at 260 and 280 nm. First-strand cDNA was synthesized using 2 µg of total RNA, a random hexamer, and PCR (Qiagen, Hilden, Germany) was performed using primers of PPARγ1 forward: (5′-CTGTGAGACCAACAGCCTGA-3′) and reverse: (3′-AATGCGAGTGGTCTTCCATC-5′); PPARγ2 forward: (5′-TTTTCAAGGGTGCCAGTTTC-3′) and reverse: (3′-AATCCTTGGCCCTCTGAGAT-5′) and internal control gene β-actin forward: (5′-AGCCATGTACGTAGCCATCC-3′) and reverse: (3′-CTCTCAGCTGTGGTGGTGAA-5′). The mixture (20 μL) for PCR included 1 μL of template, 10 μM primers (1 μL for each), 10 μL of PCR Master Mix (2X, Thermo Fisher Scientific Inc., Waltham, MA, USA), and ddH_2_O. Conditions for PCR were as follows: predenaturation at 95 °C for 10 min, 40 cycles of denaturation at 94 °C for 15 s, annealing at 60 °C for 30 s and extension at 72 °C for 30 s, and a final extension at 72 °C for 5 min on an ABI 7500 PCR system (Applied Biosystems Inc., Foster City, CA, USA). The comparative threshold cycle 2^−∆∆*C*T^ method was used to determine the expression of target genes.

### 4.5. Immunofluorescent Staining

3T3-L1 cells after 4% paraformaldehyde fixation were used to determine p-ERK (1:400 dilution; Cell Signaling, Boston, MA, USA) or HSL/p-HSL (1:400 dilution; Cell Signaling, Boston, MA, USA) protein immunofluorescence. After 8 days of culture, mature 3T3-L1 adipocytes were pretreated with KMUP-1 (10, 20 µM) for 2 or 4 days and then co-incubated with fluorescent lipid soluble dye boron-dipyrromethene (BODIPY 493/503) [34]. HSL/p-HSL and p-ERK were detected with a secondary antibody conjugated to Cy3 (red) and FITC overnight at 4 °C [34]. DAPI was used for staining nucleus in blue. All images were collected and resulting data were analyzed by confocal laser-scanning microscope (Olympus Fluoview FV1000, Olympus Optical Co., Tokyo, Japan). The ratio of average pixel intensity around the oil droplet over average pixel intensity of the cytosol was termed translocation to the oil droplet and used as an index to compare with experimental conditions or the control.

### 4.6. Reagents

KMUP-1 HCl was synthesized in our laboratory. Buffer reagents, caffeine, dexamethasone, IBMX, insulin, KT5720, KT5823, MTT (3-[4,5-dimethylthiazol-2-yl]-2,5-diphenyltetrazolium bromide), Oil Red O solution, ractopamine HCl, sildenafil citrate, simvastatin, and theophylline were purchased from Sigma-Aldrich Chemical Co. (St. Louis, MO, USA). All drugs and reagents were dissolved in distilled water unless otherwise stated. IBMX, KT5720, KT5823, ractopamine, sildenafil, and simvastatin were dissolved in DMSO at 10 mM. Serial dilutions were made in phosphate buffer solution, with a final solvent concentration of ≤0.01%.

### 4.7. Statistical Analysis

The experimental results were expressed as means ± S.E. Statistical differences were determined by one-way analysis of variance (ANOVA). When appropriate, a Tukey-Kramer pairwise comparison was used for post hoc analysis. A *p*-value <0.05 was considered significant in all experiments.
